# Finding Needles in Haystacks and Inferring Their Function: Challenges and Successes in Beneficial Symbiosis Research

**DOI:** 10.1128/mSystems.00243-21

**Published:** 2021-04-06

**Authors:** Gordon M. Bennett, Elizabeth Heath-Heckman, E. Maggie Sogin

**Affiliations:** a Department of Life and Environmental Sciences, University of California Merced, Merced, California, USA; b Department of Integrative Biology, Michigan State University, East Lansing, Michigan, USA; c Department of Molecular and Cellular Biology, University of California Merced, Merced, California, USA; d Department of Microbiology and Molecular Genetics, Michigan State University, East Lansing, Michigan, USA

**Keywords:** agricultural pest, bacteria, evolution, genome, nematodes, nutrition, symbiosis

## Abstract

Symbioses between hosts and beneficial microbes are key drivers of biological innovation and diversity. While a range of systems have emerged that provide foundational insights into how symbioses function and evolve, we still have a limited understanding of the vast diversity of organisms that engage in such interactions. Recent advances in molecular tools, theory, and interdisciplinary approaches now permit researchers to expand our knowledge and to press forward the frontiers of symbiosis research. As described in a recent issue of *mSystems*, Myers and colleagues (K. N. Myers, D. Conn, and A. M. V. Brown, mSystems, 6:e01048-20, 2021, https://doi.org/10.1128/mSystems.01048-20) conducted a genome skimming approach to understand the role of obligate beneficial symbionts in plant-parasitic dagger nematodes. Nematodes are extraordinarily abundant and key players in ecosystem function and health. However, they are difficult to harness in the lab. The approach used by Myers et al. ameliorates these challenges to illustrate a relatively complete picture of a poorly understood beneficial symbiosis.

## COMMENTARY

Alliances with microbes enable eukaryotic hosts to extend their phenotypes beyond their own genomic encoded capabilities (1, 2). From single-celled amoebae and fungi to plants and animals, beneficial microbes generally allow their hosts to exploit environments typically out of bounds for their independent survival (3–5). In recent years, our understanding of such symbiotic interactions has dramatically shifted due to nothing short of a modern-day technological renascence (6). Novel biological insights from a growing range of symbiotic systems have been enabled by the development and application of advanced molecular tools, basic theory, and interdisciplinary science (7). Yet, despite our growing understanding of the diversity and importance of beneficial symbioses in the biological world, several key challenges still exist in this field.

First, the broad biological and evolutionary diversity of beneficial symbioses is still poorly characterized. The recent development of model systems for understanding symbiotic interactions has provided foundational theory to guide scientific inquiry (8). However, while expectations and tools from these systems can be—and have been—successfully applied to a wider range of organisms (9), basic investigation of the symbiotic interactions among the vast diversity of organisms is in many ways still lacking. Beneficial symbioses are likely so widespread in nature that we have only begun to scratch the surface of their true abundance and diversity. For example, many small and microscopic invertebrate animals that live in complex environments (e.g., soils and oceans), or that are themselves host dependent, ally with beneficial bacterial symbionts (10, 11). But, due to compounding limitations in technological and human resources, much remains unknown about these intriguing symbiotic systems. Without a complete understanding of the range of symbiotic interactions that exist in nature, let alone more fine-grained insights into how they function, it is difficult to develop fully integrated and comprehensive theories for this field of biology.

Second, it can be challenging to correctly identify the key microbial players and their functional roles within a beneficial symbiosis. Given that all hosts live in a world dominated by microbes, disentangling which, if any, microbes play essential roles in a host is far from straightforward (e.g., see reference 12). Many candidate microbes may have high prevalence within and among host populations, but do not confer essential benefits and may even be parasitic (13). Moreover, hosts may obligately rely on a complex consortium of symbionts that provide distinct, but complementary, essential services (14). In these cases, genomic sequencing and metagenomic approaches provide opportunities to jigsaw together the individual contributions and requirements of each partner (15). Paired with basic theory and hypotheses of the putative roles that symbionts can play (e.g., defense and nutrition), researchers should be able to capably infer their functional roles based on the genes and metabolisms retained in their genomes. Indeed, such approaches have helped establish foundational evolutionary and biological theory for a diverse set of symbiotic organisms (16, 17).

Third, even though ecological, evolutionary, and genomic approaches can generate key hypotheses of how beneficial symbioses function, specific tests of symbiotic physiologies are often prohibitively difficult. Microbial symbionts that become restricted and dependent on host environments cannot easily be cultured, rendering direct experimentation challenging, if not impossible (18). In extreme cases, their genomes become so reduced and metabolically limited that they cannot synthesize even the most basic and essential cellular resources, including cell membranes, energy, basic nutrition, etc. (19). While advances in metabolic modeling may make culturing and harnessing of some symbiotic microbes a future possibility (7), many interested organismal biologists may not have the training or resources to do so. An alternative approach is to use the host itself as the “petri dish” to test predictions of physiology. Recent work in several well-studied symbiotic systems have ushered in an exciting new era through the interdisciplinary application of cutting-edge molecular, microbiological, and bioengineering approaches to test functional hypotheses (e.g., see references 20 to 24). While these tools generally require tailoring to a specific system, they highlight a promising future by holistically leveraging the host itself to reach a gold standard of *in vivo* tests of function.

In their recent paper, Myers and colleagues (25) tackle two of the three research challenges outlined above via a comparative evolutionary genomics framework. Their aim is to understand symbioses in tiny soil-dwelling, plant-parasitic dagger nematodes (*Xiphinema* spp.). Nematodes are microscopic animals that can be challenging to work with under laboratory conditions (25, 26). They are profoundly diverse and abundant, playing key roles in soil health, plant and animal disease, and ecosystem function (27). Given the diversity and ubiquity of nematodes, many species likely rely on microbes for beneficial phenotypes. Recent studies have indeed confirmed that several plant- and animal-associated nematode species rely on obligate associations with bacteria for essential nutrition limited in their host niche (11, 25, 28). However, identifying nematode-associated symbionts and elucidating their functional roles is far from straightforward. In their paper, Myers et al. use next-generation sequencing approaches to skim the bulk genomes of nematodes and their potential symbionts out of complex environmental soil samples. This approach obviates the need to isolate and culture individual nematode species. From their data, the authors are able to identify and pair host-symbiont diversity, assemble complete bacterial genomes, and use population genetic diversity metrics to understand how evolutionary pressures shape the evolution of symbiotic bacteria.

The specific findings of Myers and colleagues paint a comprehensive picture of a beneficial microbial symbiosis in dagger nematodes. Some *Xiphinema* species are thought to obligately parasitize plant vascular tissues and phloem—a specialized diet that is potentially limited in essential nitrogen, indicating that a symbiosis may be afoot. Indeed, sampled *Xiphinema* consistently associate with a *Xiphinematobacter* bacterium. This *Xiphinematobacter* species has a relatively tiny genome compared to that of its free-living relatives, which is a key characteristic of ancient host-associated microbes (29). Its highly streamlined genome is enriched in nutrition synthesis pathways, including essential amino acids and B vitamins that animals are incapable of making. In this context, Myers et al. present a convincing theory that at least some *Xiphinema* species intern *Xiphinematobacter* that extends their phenotype through the provisioning of essential nutrition lacking in their specialized plant diets. Beyond the basic genomic characterization of this bacterium, their genetic data also contain population genomic information. These data illustrate that selection maintains the symbiotic nutritional role of *Xiphinematobacter* and its persistence across its nematode hosts.

Taken together, Myers and colleagues provide a framework for pursuing an integrative understanding of beneficial symbiosis in understudied biological systems. Their data provide a treasure trove of information that tests questions of microbial symbiont identity, diversity, function, and evolution. Their results fit nicely within, and also expand, the existing theory of beneficial symbioses by providing a clear description of a poorly understood system (17). Genomic studies, as illustrated by Myers et al. (25), are critical to developing foundational knowledge of eukaryote-microbe interactions. They also lay the essential groundwork to test key physiological predictions of how such tightly entwined systems are functionally integrated and maintained.
